# Foreign Nationality, Family Psychiatry History and Pregestational Neoplastic Disease as Predictors of Perinatal Depression in a Cohort of Healthy Pregnant and Puerperal Women during the COVID-19 Pandemic

**DOI:** 10.3390/healthcare11030428

**Published:** 2023-02-02

**Authors:** Laura Orsolini, Simone Pompili, Antonella Mauro, Umberto Volpe

**Affiliations:** Unit of Clinical Psychiatry, Department of Neurosciences/DIMSC, Polytechnic University of Marche, 60126 Ancona, Italy

**Keywords:** perinatal depression, postpartum, predictors, pregnancy

## Abstract

**Background:** Perinatal depression (PND) represents one of the most common mental disorders in the pregnancy and/or postpartum period, with a 5–25% prevalence rate. Our aim was to investigate predictors associated with PND in a cohort of pregnant and puerperal women based in an Italian setting during the COVID-19 pandemic. **Methods:** We retrospectively recruited 199 (55 pregnant and 144 puerperal) women, afferent to our Perinatal Mental Outpatient Service of Ancona (Italy). Participants were administered an ad hoc case-report form, Whooley Questions (WQ), the General Health Questionnaire-12 (GHQ-12), the Stress Holmes-Rahe scale (HR) and the Edinburgh Postnatal Depression Scale (EPDS). **Results:** Around 10% of the sample had a confirmed PND. Being a foreigner woman (RR = 3.8), having a positive psychiatric family history (RR = 5.3), a pre-pregnancy medical comorbidity (RR = 1.85) and a comorbid medical illness occurring during the pregnancy (RR = 2) were much likely associated with PND. Multiple linear regression analysis demonstrated that GHQ, medium- and high-risk at the HR, foreign nationality, positive family psychiatric history, and neoplastic disease before conception significantly predicted EPDS [*F*(1, 197) = 10.086, *R*^2^ = 0.324, *p* < 0.001]. **Limitations:** The sample size, poor heterogeneity in terms of socio-demographic, clinical and gynecological-obstetric characteristics, the cross-sectional design of the study. **Conclusions:** Our study showed a set of predictors associated with a higher risk for the PND onset, including gestational and pregestational medical disease. Our findings outline the need to screen all fertile women, particularly in gynecological and medical settings, in order to identify at-risk women for PND and promptly suggest a psychiatric consultation.

## 1. Introduction

The perinatal period (i.e., the span throughout the pregnancy until the first postpartum year) is a critical and vulnerable time in which women may experience high psychosocial distress [1,2]. According to the bio-psycho-social paradigm of mental disorders [3], pregnant and puerperal women have to face significant psychological, biological, physical and social changes [1,2,3] that can lead to de novo onset or recurrence of mental health conditions [4,5]. Perinatal depression (PND), which includes major and minor depressive episodes occurring during pregnancy or in the first 12 months after delivery [6], is one of the most common mental illnesses developing during the perinatal period [7,8]. The prevalence of PND varies from 5% to 25% [9,10,11]. This wide variation in prevalence rates depends on several factors, such as methodological issues, the geographical setting (high- vs middle/low-income countries), or the gestational period considered [10,11]. Indeed, a systematic review and meta-regression including 101 studies, found a PND prevalence rate of 11.9% in high-income countries [11]. Moreover, a very recent study reported that the prevalence of PND ranges from 11.3% to 19.6% during pregnancy, while from 9.6% to 24.3% during the postpartum period [12]. Several risk factors have been investigated to be implicated to PND [4], among these having a previous history of a psychiatric disorder is that more frequently associated with PND onset [13,14]. Moreover, the likelihood to be hospitalized due a psychiatric disorder is reported to be around 22-fold more higher during the first postpartum month, compared to pre-pregnancy period, particularly in women with a pre-existing severe mental illness [15,16]. Furthermore, specific sociodemographic, psychological and cultural factors have been associated with PND, such as the lack of social support, poor family economic status, unplanned pregnancy, lower maternal educational levels, unemployed and financial problems, adverse life events (i.e., domestic violence, physical or sexual abuse), previous negative pregnancy experience and the fear of the delivery [4,13,14,17,18]. Personality characteristics such as pessimism, self-criticism, low self-esteem, high need for achievement and strong emotional dependence towards other people are considered risky factors for developing depressive disorders in the perinatal period [17,19]. In addition, other studies investigated the impact of medical diseases on mood disorder, also in the PND [20,21]. A recent meta-analysis found an association between hypertension, diabetes mellitus (DM), heart disease, migraine, and other neurological disorders with the risk of developing PND [20]. Obesity, premenstrual syndrome (PMS), DM, thyroid dysfunction, HIV infection, polycystic ovary syndrome (PCOS) and other illnesses have been suggested to increase the likelihood of PND [18,22,23,24]. Similarly, other studies demonstrated the impact of gestational comorbidities and perinatal complications experienced by women during pregnancy and delivery on mood disorders, including PND [18]. Gestational diabetes, anemia during pregnancy, vitamin D deficiency, preterm birth and postpartum anemia would seem to be risky factors for developing PND [18,25]. Moreover, previous research found that perinatal complications can lead to perinatal mental disorders, and similarly, pre-existing mental disorders can increase the risk of perinatal complications [5]. Furthermore, untreated mental disorders can negatively affect both maternal and child health [26,27]. However, only few studies have investigated the prevalence and the predictors for PND in an Italian sample [7,9,28,29,30] and no studies based in Italian settings, more specifically addressed potential predictors of PND in a sample of healthy pregnant and puerperal women (without a previous psychiatry history) during the COVID-19 pandemic. Therefore, given limited data available regarding the potential predictors of PND in a sample of healthy pregnant and puerperal women, in Italian settings and during the COVID-19 pandemic, and particularly focusing on the concomitant medical diseases, both those already pre-existing during the preconception period and those occurring de novo during the pregnancy, we retrospectively collected a sample of pregnant and puerperal women afferent to our Italian Peripartum Psychiatry Outpatient Service within a regional screening program, among those hospitalized at the Unit of Clinical Gynecology and Obstetric of our same university hospital. The main objectives were: (a) investigating the prevalence of PND in our sample of pregnant and puerperal women, assessed during their third trimester of pregnancy and during their first trimester of postpartum period, and without a previous history of a psychiatric disorder during the Italian COVID-19 pandemic; and (b) identifying (if any) sociodemographic, clinical and obstetrical features could act as predictors for the development of a PND in a healthy sample of pregnant and puerperal women in an Italian hospital setting.

## 2. Materials and Methods

### 2.1. Study Design and Selection of Participants

The present study is part of a larger multicenter nationwide population-based naturalistic observational screening project aimed at implementing diagnostic and therapeutic approaches for early detection and treatment of women at-risk for developing perinatal mental disorders. A chart-review study was conducted by retrospectively recruiting all women afferent to the Peripartum Psychiatry Outpatient Service of the Unit of Clinical Psychiatry at the University Hospital “Ospedali Riuniti”, Polytechnic University of Marche, Ancona, Italy, among those women hospitalized at the Unit of Clinical Gynecology and Obstetric at the University Hospital “Salesi”, Ancona, Italy, between December 2020 to June 2021. Written informed consent for research purposes was obtained from all participating women. All women were given the possibility to withdraw their participation from the study, without any kind of clinical and therapeutic consequences. Recruitment and enrollment of the final sample were based on the following inclusion criteria: (a) ≥18 years old; (b) absence of linguistic difficulties (i.e., not fluent Italian speaker and/or without a sufficient ability to understand Italian language); (c) pregnant women or within their first postpartum year; (d) no intellectual disability or cognitive impairment; (e) women without a previous psychiatric illness (before the pregnancy); (f) signed informed consent for collecting and analyzing clinical data for research purposes, collected during the baseline assessment. Participants were excluded if they met one or more of the following exclusion criteria: (a) being under the influence of substances and/or alcohol; (b) incomplete or inadequate filled out questionnaires; (c) refusal to complete the informed consent. All the study procedures were in accordance with the ethical standard of the institutional and/or national research committee and with the 1964 Helsinki Declaration and its later amendments or comparable ethical standards. The institutional review board approved the study. This research study was conducted retrospectively from data obtained for clinical purposes.

### 2.2. Measures

All participants were asked to complete an ad hoc case report, specifically designed by the researchers, to collect sociodemographic data (e.g., age, ethnic, marital status, employment status, education level) and clinical and pregnancy-related data (e.g., familial psychiatric history, medical history, body mass index (BMI) before pregnancy, thyroid-stimulating hormone (TSH) levels before the pregnancy including in the preconception laboratory tests), obstetric-gynecologic variables such as gestational comorbidities, previous miscarriage/induced abortion, pregnancy course, delivery course and perinatal outcomes. According to the 2014 National Institute for Health and Care Excellence (NICE) [31] guidelines on antenatal and postnatal mental health, all women were administered the Whooley’s questions [32,33], the General Health Questionnaire (GHQ-12) [34,35], the Stress Scale by Holmes and Rahe [36] and, as screening tool for detecting depressive symptomatology in pregnancy and during the postpartum period, the Edinburgh Postnatal Depression Scale (EPDS) [37,38]. The clinical diagnosis of PND was carried out by senior psychiatrists through a semi-structured clinical interview for DSM-5 (SCID-5-CV) [39].

The Whooley items [32,33,40] consist of the following two case-finding questions: (a) “During the past month, have you often been bothered by feeling down, depressed or hopeless?”; (b) “During the past month, have you often been bothered by having little interest or pleasure in doing things?”, aimed to identify potential low mood and loss of interest or pleasure during the past month [32,33]. The Whooley questions are recommended by NICE as a primary index test to identify depression during the perinatal period [31]. Whether a woman answers yes to either question should be addressed to a psychiatric consultation for investigating a possible PND [41]. The sensitivity and specificity of the Whooley questions were estimated in the range of 46–100% and 65–92%, respectively [42].

The GHQ-12 is a 12-item scale consisting of four seven-item subscales measuring social dysfunction, health perception (somatic symptoms), anxiety/insomnia and severe depressive symptoms, during the preceding 2 weeks [35]. Each subscale has seven questions and each item has four optional responses scored 0 to 3 as follows; score 0: “not at all” score 1: “no more than usual”; score 2: “rather more than usual” and score 3: “much more than usual.” The total score can range from 0 to 36 points. Higher scores indicate greater impairment, with a set cut-off higher than 21 for the Italian sample (Cronbach’s alpha of 0.85) suggestive of a mental health distress [43].

The Holmes and Rahe Stress Scale [36] consists of 42 vital events, such as the death of a spouse or a close relative, divorce, marital separation, professional changes, among others, to which a score is assigned according to the stress it may have generated to the individual. The scale analyzes the difficulty required for the person to readjust to society after significant changes in their life, which generate emotional distress leading to various diseases. The instrument measures the intensity and duration of the time needed to adapt to a life event and is based on the concept that any change is considered a stressful factor. Each event has a score given by the authors of the instrument, ranging from 11 to 100 points. The Holmes and Rahe Stress scale were categorized into three groups for risk of disease development associated with chronic stress levels: low risk (LR) (final score lower than 149), medium risk (MR) (final score 150–299) and high risk (HR) (final score greater than 300) [36].

The EPDS is a 10-item self-report scale, widely used in research in perinatal mental health [44,45] and it has been validated for use in all perinatal period [46,47,48]. EPDS measures the severity of depression symptomatology experienced by the woman over the previous seven days and it has been proven to be an efficient and effective tool for early identification of women at-risk for PND [6]. Each item is scored on a four-point Likert scale (0–3), with a total score ranging from 0 to 30, and a clinically cut-off of ≥12 for the Italian sample which denotes a higher risk for developing PND [49]. The Italian version of EPDS showed good sensitivity (55.6%), high specificity (98.9%) and high positive predictive value (90.9%) [50]. In our study, cut-off of ≥12 at the EPDS was used to early identify patients with PND, according to the previous published literature and international guidelines [6,29,30,31,50,51].

### 2.3. Statistical Analysis

Categorical variables (i.e., sociodemographic features, clinical and pregnancy-related variables) were presented by frequencies (n) and percentages (%). Qualitative variables, whereas normally distributed, were expressed as mean and standard deviations (SD); whereas not normally distributed, as median and 95% confidence interval (CI). After analyzing the continuous variables for skewness, kurtosis, normality distribution through the Shapiro-Wilk test, and the equality of variances by Levene test, parametric or non-parametric statistical tests were used when appropriate. One-way analysis of variance (ANOVA) or Kruskal-Wallis tests were used, where appropriate, to compare EPDS total scores according to all sociodemographic, obstetric and psychopathological variables. The sample was also divided in two groups according to the EPDS total score: those women positive to the PND screening (EPDS+) and those women without significant EPDS scores (EPDS-), in order to identify possible risk factors for the development of PND. The χ^2^ Test was used to examine differences in the distribution of all sociodemographic, obstetric and psychopathological variables between two groups (EPDS+ vs. EPDS-). Multiple linear regression analyses were performed to examine the linear relationship between EPDS scores (as dependent variable, outcome) and all socio-demographic and clinical variables (as independent variables, predictors). For this purpose, a backward selection for the multiple regression model was run for each explanatory variable. The selection process stopped and was accepted at the resulting model when all variables in it were statistically significant. The level of significance was set at *p*-value less than 0.05. Statistical analysis was performed using SPSS version 26 for MACOS (IBM Corp, Harmony, Armonk NY, USA, 2019).

## 3. Results

### 3.1. Socio-Demographic Features of the Sample

All socio-demographic and clinical characteristics of the included subjects are summarized in Table 1 and Table 2. A total of 244 women were screened in the timeframe December 2020–June 2021. Among these, 29 patients did not fill the questionnaire correctly, 13 subsequently decided to withdraw from the study and three women voluntarily discontinued the pregnancy. Therefore, a final sample of 199 subjects was finally recruited in the present study, of which 144 women were in their postpartum period while 55 women were recruited during their pregnancy. The mean age of the women was 34.0 (SD = 4.9). The education level, expressed as the mean number of study years, was 14.6 (SD = 2.8). Most of the sample was Italian (N = 179; 90%), married or cohabiting with a partner (N = 182; 91.5%) and full and/or part-time employed (N = 168; 84.4%) (Table 1).

### 3.2. Clinical and Psychopathological Features of the Sample

According to the Whooley questions, around 20.1% of the sample (N = 40) was positive to the screening questions for a possible PND. The mean GHQ total score was 13.8 (95% CI = 13.1–14.6), with a significant mental distress reported in 8% of the sample (N = 16). The median HR total score was 122.2 (95% CI = 109.1–135.2), being found a medium risk for stress-related mental disorders in 25.6% (N = 51) of the sample while a high risk in 4% (N = 8) of the entire sample. The mean EPDS total score was 5.8 (SD = 4.8), with a 10.1% prevalence of the recruited women who reached a significant EPDS total score, indicative of PND.

The differences in sociodemographic and clinical variables between the two subgroups (EPDS+ versus EPDS-) are summarized in Table 1 and Table 2. The differences in gynecological-obstetric variables between the two subgroups (EPDS+ versus EPDS-) are summarized in Table 3; while delivery-related features in Table 4. Most participants showed, before the conception, a normal weight (N = 134; 67.3%) and normal levels of TSH (N = 165; 82.9%) (Table 2). A concomitant preconception medical illness was reported in 30.7% of the sample (N = 61) (Table 2); while 42.7% of the sample (N = 85) developed a medical comorbidity during the gestational period (Table 3). Approximately 35.2% of the sample (N = 70) declared a concomitant medical pharmacological treatment and the 30.7% of the sample (N = 61) reported a positive family psychiatric history (Table 2). 

### 3.3. Clinical and Psychopathological Predictors of PND

Statistically significant higher EPDS scores were found among women with a foreign nationality compared to the Italian women (*p* = 0.008), with a higher risk to develop PND (relative risk [RR] = 3.8, 95% confidence interval [CI] = 1.7–8.9). A positive psychiatric family history is associated with significantly higher EPDS scores (*p* < 0.001) and a higher risk to develop a PND (RR = 5.3, 95% CI = 2.1–13.1). Regarding pre-pregnancy medical comorbidities, women affected by preconception medical illnesses displayed significantly higher EPDS total scores (*p* = 0.014), compared to healthy women (RR = 1.85 (95% CI = 0.8–4.2). Among medical illnesses, endocrinopathy (*p* = 0.022) was significantly associated with PND (RR = 3.4 [95% CI = 1.4–8.2]), as well as having a history of neoplastic disease (*p* = 0.014) (RR = 5.7 [95% CI = 2.3–14–2]) (Table 3).

Similarly, women with a comorbid medical illness occurring during the pregnancy showed significantly higher EPDS scores (*p* = 0.015), compared to those women without a comorbid medical disease during the pregnancy (RR to develop a PND = 2.0, 95% CI = 0.9–4.7). In particular, gestational hypertension, with or without preeclampsia (*p* = 0.030) and the occurrence of shortening of the uterus neck (*p* = 0.049) were significantly associated with higher EPDS scores. The RR for developing a PND is 2 (95% CI = 0.7–6.2) for gestational hypertension and 3.7 (95% CI = 1.3–10.4) for shortening of the uterus neck. Women with a previous history of miscarriage reported significantly higher EPDS scores (*p* = 0.007), with a RR for developing a PND of 2.1 (95% CI = 0.9–4.8) of developing a PND (Table 3).

No significant differences were observed between the two groups regarding the age, the level of education, the employment, previous delivery, the type of conception (physiological versus medical-assisted), BMI ≥ 25, and the value of TSH (Table 2 and Table 3). No significant differences were found either for the delivery-related variables, such as the time and type of delivery, Apgar score, birth weight, cranial circumference at birth, cranio-caudal length and type of breastfeeding (Table 4).

Linear regression analysis found that GHQ scores [*F*(1, 197) = 5.478, *R*^2^ = 0.027, *p* = 0.020] and HR scores [*F*(1, 197) = 39.897, *R*^2^ = 0.168, *p* < 0.001] statistically significantly predicted EPDS scores. A positive correlation was found between GHQ (*r* = 0.164) and HR (*r* = 0.410) and EPDS. A multiple linear regression analysis demonstrated that all the socio-demographic and clinical variables, statistically significant at ANOVA (i.e., positive screening at the Whooley questions, positive mental distress at the GHQ, medium- and high-risk at the HR, foreign nationality, positive family psychiatric history, neoplastic disease before conception, gestational hypertension/preeclampsia, miscarriage threats and/or placental abruption, and shortening of uterus neck), significantly predicted EPDS total scores [*F*(1, 197) = 10.086, *R*^2^ = 0.324, *p* < 0.001], except for the presence of gestational hypertension/preeclampsia, miscarriage threats and/or placental abruption, shortening of uterus neck and a positive screening at the Whooley questions (Table 5).

## 4. Discussion

Our findings showed a clinically relevant PND in 10% of the sample, as measured by EPDS, a percentage within the range already described by previous international studies which reported a PND prevalence ranging from 10% to 20% [11,28,52,53]. Moreover, our result is also within the Italian PND prevalence, already reported by previous studies, ranging from 2.2% to 26.6% [7,9,28,54,55,56]. Notably, despite our findings having been collected during the COVID-19 pandemic in Italy, our estimated PND prevalence is lower than other studies carried out in Italian settings during the pandemic period which reported a prevalence ranging from 28.6% to 46% [57,58]. However, a possible explanation could be that our participants have been recruited only by those who did not have a previous psychiatry history. Another possible reason could be that most of the studies were conducted during the first waves of the COVID-19 pandemic [51,59,60,61]. Therefore, one could argue that the unprecedented and unpredictable situation of the early phases of COVID-19 pandemic, associated with the increased fear of contagion, specifically for their own and their newborn’s health consequently to COVID-19 infection, together with mental consequences following COVID-19 lockdown and relative restrictions could have determined an increased PND prevalence during the early phases of the COVID-19 pandemic. While, our sample was mainly collected during the later phases of the Italian COVID-19 pandemic, hence, by describing a picture of the third wave of Italian COVID-19 pandemic.

Our findings identified a set of sociodemographic and clinical risk factors, significant predictors for the development of PND in our sample. PND women were mainly represented by foreign women. In this regard, a large amount of literature already documented that the immigration status may increase the PND risk [13,62]. In fact, foreign mothers have to face multiple psychological and socio-economical stressors before, during and after migration that can lead to depressive disorders [63], which could be potentially amplified during pregnancy and/or postpartum [64]. Immigrant pregnant and/or puerperal women could often experience social isolation, lower socioeconomic status, and the loss of family support and social network during a period of extreme psychological and emotional vulnerability [63,65]. The lack of a family, economic and social support could also limit their access to health care facilities, mainly due to the linguistic and cultural barriers [66]. Moreover, even though not specifically evaluated in our study, it has also been well documented that interpersonal violence and a history of trauma, are often frequently reported among immigrant and/or refugee women, which in turns may determine a higher risk for developing moderate-to-severe PND [65]. While our study did not find significant differences in PND, according to the marital status. In fact, previous studies reported that the single mother status could represent a significant risk factor for developing PND [67,68,69]. Accordingly, further studies already documented that PND women were more likely to be not married, single or have partners not living in the same household [67,68,69]. Single mothers may be afraid of being unable to take care of their child, display more difficulty in finding work and, consequently, may have financially precarious situations [70]. Moreover, in some cultures they have to face the social stigma of being unmarried [67]. Furthermore, several studies confirmed that having a perceived family and/or social support and a marital satisfaction are, indeed, protective factors for the development of a PND [69,71]. While conflictual relationships with the partner represent a risk factor for the onset of PND [9,72]. Likewise, a lack of social support has been widely and strongly associated with poor maternal mental health and PND [68,73,74]. Moreover, no significant associations were found between women’s age, economic status and education level and PND in our study, contrarily with previous literature [62,75,76]. In fact, younger (<20 years) maternal or advanced age (>35 years), lower socioeconomic status, low educational status and unemployment have been previously demonstrated to be significant predictors for the onset of a PND [17,71,76,77,78]. However, these data could greatly depend on the differences in ethnicity, socio-cultural factors and the geographic origin of pregnant and/or postpartum women recruited in previous studies [17,77]. In fact, one could argue that our sample could not be sufficiently representative of all perinatal women, being mainly constituted by participants with moderate-to-high cultural, educational and economical levels.

Furthermore, our findings confirmed a significant association between PND and a family psychiatric history, in line with previous literature [28,74,77]. This is an interesting finding, considering that our sample is constituted by pregnant and puerperal women without a previous personal psychiatry history. The familiarity of PND has been indeed already well investigated in other studies which documented that daughters of prenatally depressed women display over 3-fold higher risk of developing PND during pregnancy, compared to those women without a positive family history for PND [13,79]. Furthermore, the genetic aspect of the PND is still under investigation [80] (Border et al., 2019). Different pathways are still being studied, such as the expression of CLOCK genes, oxytocin and oxytocin receptor (OXTR) genes, single-nucleotide polymorphism (SNPs) related to stress regulatory genes or estrogen/serotonergic receptors, as well as genes related to hypothalamic-pituitary-adrenal axis (HPA) or inflammatory system [81,82,83]. However, there are no specific candidate genes or genes clearly related to PDN yet [80], despite promising studies coming from the polygenic risk score (PRS) and/or the epigenetic phenomena [84].

Furthermore, our sample found that PND women significantly displayed higher rates in comorbid chronic medical conditions, such as neoplastic and endocrinological diseases, compared to non-PND women, consistent with previous studies [68,85,86,87,88,89]. In this regard, according to a recent systematic review and meta-analysis, including 16 studies comprising 1,626,260 women, individuals with chronic medical condition, such as diabetes, hypertension, heart disease, migraine and other neurological disorders, displayed a higher risk for developing a PND [20]. However, our regression analysis did not confirm the significant association between PND and comorbid endocrinopathies while confirming the role of neoplastic diseases that occurred before the conception in significantly increasing the risk for PND development. In addition, to the best of our knowledge, our study is the first to report a significant association between a neoplastic condition and a higher risk for PND. However, this finding could be supported by previous evidence and studies reporting a strong association between the occurrence of depressive symptomatology and the neoplastic condition among women in general, independently by the status of pregnancy and/or postpartum [90,91].

Furthermore, our study investigated the possible relationship between baseline TSH levels before the conception and depressive symptoms without finding any statistically significant association. The rationale of this initial investigation was derived by the evidence that some pregnant women may experience thyroid morphology changes and/or thyroiditis that can be accompanied by depressive symptomatology [22] and that there is a strong association between a thyroid dysfunction and the occurrence of a MDD [92,93,94]. However, previous studies have already investigated TSH and thyroid peroxidase antibodies (TPO-ab) levels as possible biomarkers for PND, by indeed describing inconclusive results [22].

Furthermore, our findings found that a comorbid pregnancy-related disease could be a significant predictor for the onset of PND, as previously documented [20,68,76]. In particular, our study found that gestational hypertension, with or without preeclampsia, shortening of uterus neck, a history of miscarriage threats and/or placental abruption, were significant associated with higher EPDS total scores, even though multiple linear regression analysis did not confirm them as potential predictors for PND. Literature so far available already documented that women with hypertensive diseases during the pregnancy (HDP), including chronic hypertension, gestational hypertension and preeclampsia (PE), more likely displayed the occurrence of depressive symptomatology, compared to healthy controls [95,96]. Moreover, several studies found that PE seemed to be independently associated with PND, being the risk indeed directly associated with the worsening of PE [25]. A previous study by Youn et al., (2017) [76] also reported that an experience of placental abruption or PE were significant predictors for the onset of a PND, coherently with our findings. In addition, to the best of our knowledge, our study was the first to report a significant association between PND and the shortening of the uterus neck. While our study did not identify a significant association between a history of previous miscarriage and primiparous status and the occurrence of a PND, in disagreement with previous literature [17,97,98,99,100,101,102,103,104]. However, also other studies did not find any significant association between spontaneous or induced abortion and PND [105,106,107]. In addition, we did not find any significant association between a set of perinatal obstetric variables (i.e., preterm birth, low birth weight, cesarean delivery, low Apgar score and decrease in breastfeeding intention) and the onset of a PND, contrarily with previous studies [85,108,109,110,111]. A possible explanation could depend on the different methodology of the studies, as well as in the adopted definition of perinatal obstetric complications [112]. Moreover, our study did not observe an association between obesity and PND, whereas recent studies documented this relationship [113,114].

Overall, PND is often underdiagnosed and undertreated [115,116], mainly due to the social stigma experienced by women who explicitly manifest depressive symptoms during pregnancy and/or postpartum period [17,117,118] or because women could be more reluctant to seek mental health help rather than for a physical problem [119]. Furthermore, clinicians (particularly gynecologists and/or general practitioners) could not be adequately trained to early identify atypical symptoms (i.e., fatigue, loss of energy, appetite and sleep change) which can accompany a depressive state in the perinatal period and, hence, the condition could be often underdiagnosed and under-evaluated [120,121]. However, an untreated PND is often associated with negative outcomes both for the mother and the newborn, a deficient mother-infant bonding, and neurodevelopment disorders in the newborn [26,27,122]. Furthermore, maternal depression represents one of the leading causes of maternal mortality in pregnancy and postpartum [123,124]. Therefore, understanding which factors/predictors are potentially associated with a higher risk for developing PND should be a clinical and public mental health priority, in order to implement preventive strategies approaches to reduce/eliminate associated risk factors and adequately treat PND women and those at-risk for developing PND. In particular, according to our preliminary findings, particular attention should be paid to immigrant women, single mothers, patients with a family positive psychiatric history, pregnant and/or postpartum women affected with chronic medical illness and/or gestational medical comorbidities, as these factors may act as predictors for the development of a PND (Biaggi et al., 2016). Indeed, PND is a result of a complex interaction involving genetics, epigenetic, dysregulation of hypothalamic-pituitary-adrenal (HPA) axis, increase in inflammatory response, alteration of circadian rhythms, several environmental and social factors [17,81,88,110,125]. Therefore, implementing interventions to support pregnant and/or postpartum women with at-risk predictors for the onset of a PND, could help in reducing the impact of PND on women. Evidence-based treatments in pregnancy and postpartum period for PND women include psychoeducation, social support group, cognitive behavioral therapy (CBT), interpersonal psychotherapy (IPT), directive counseling and pharmacotherapy [6,31,115,126,127,128,129]. These interventions should comprise partners and family members of PND women [108]. Indeed, emphasizing the importance of social support during the perinatal period may favor maternal mental health outcomes [73,130]. Moreover, the health care system should facilitate access to health services for immigrant women providing programs of social support that also involve partners, interpreters and translated screening tools [88].

However, despite the promising findings, our study displays several limitations that should be adequately discussed. Firstly, the small sample size and the poor socio-demographic heterogeneity variability of the recruited sample (in terms of educational level, socio-economic and employment status) as well as psychopathological/clinical poor variability (in terms of family and/or personal psychiatry history) could limit the generalizability of our findings to the general population. Furthermore, the confounding effect of compliance bias could limit the generalizability of the present findings. Moreover, most women were recruited during their third trimester of pregnancy and during their first postpartum trimester, not making the sample fully representative of the full peripartum period. In addition, our sample has been recruited during the Italian COVID-19 pandemic, mainly during the third Italian wave, therefore, our findings could not fully represent the early phases of the COVID-19 pandemic either not necessarily being represented of not-COVID-19-related periods. Secondly, the cross-sectional study design did not allow to longitudinally monitor these clinical predictors over the time, according to the balance/unbalance between risk and protective factors, the gestational and/or postpartum period, and so forth. Thirdly, although the sensitivity of the Italian version of EPDS (Benvenuti et al., 1999) can be considered satisfactory (55.6%), being a self-report tool, the result can be voluntarily influenced by women who do not feel to be confident in reporting their depressive symptoms for many reasons, such as social stigma, fear of the possible consequences of a positive screening test, and so forth. For this reason, the use of EPDS only could determine a higher rate of false negative women. Fourthly, foreign patients without a good Italian language level were excluded from the study. This may be a limitation considering the literature. Fifthly, our study did not adequately assess the immigration status of women, the history of a previous trauma and/or interpersonal violence which could help better understand which variables may influence the most vulnerability of migrants and refugee women to PND. Therefore, further larger and longitudinal studies should be carried out to systematically investigate and confirm our preliminary findings by recruiting more Italian centers, as well as by including more heterogeneous samples, including an ethnic, educational, cultural and employment variability in the sample.

## 5. Conclusions

Perinatal period indeed represents a period of greater emotional and psychological vulnerability for the development of mental disorders, including PND. PND is a multifactorial and complex disorder in which several risk and protective factors can mutually relate to each other and influence the course of illness during the pregnancy and the postpartum period. Our findings identified a set of significant socio-demographic, clinical and psychopathological predictors which may determine a higher risk for the development of a PND. Our results suggest that special attention should be given to immigrant women, single mothers, patients with a family positive psychiatric history, pregnant and/or postpartum women affected with chronic medical illness and/or gestational medical comorbidities. Overall, all women should carefully be early screened during their perinatal period, in order to early identify those women at-risk for PND and promptly manage appropriate interventions. In particular, the screening activity should involve both gynecologists and psychiatrists. Gynecologists should be adequately informed about clinical and gynecological predictors which could significantly predispose pregnant and/or postpartum women to the onset of a PND and promptly recommend them to seek for mental health aid. Similarly, psychiatrists should properly collaborate with gynecologists in informing them about the known risk factors associated with PND onset and provide a tailored approach.

## Figures and Tables

**Table 1:** Socio-demographic characteristic of the sample.

	Total Sample (N = 199) N (%) M (SD)	EPDS- (N = 179) N (%)	EPDS+ (N = 20) N (%)	*p*-Value *	EPDS M (SD)	*p*-Value **^,^***
**Age** (year)	34.0 (4.9)	34 (4.9)	33.2 (5.6)	n.a.	-	t = 0.726
						*p* = 0.475
**Level of education** (year)	14.6 (2.8)	14.7 (2.7)	13.4 (2.9)	n.a.	-	t = 1.990
						*p* = 0.059
**Nationality**						
Italian	179 (90%)	165 (92.2%)	14 (7.8%)	χ^2^ = 9.789	5.5 (4.4)	F = 2.304
Non-Italian	20 (10%)	14 (70%)	6 (30%)	***p* = 0.008**	8.3 (7.2)	***p* = 0.002**
**Marital status**						
Married/Cohabiting	182 (91.5%)	163 (89.6%)	19 (10.4%)	χ^2^ = 0.357	4.3 (4.1)	F = 0.685
Single Status	17 (8.5%)	16 (94.1%)	1 (5.9%)	*p* = 0.470	5.9 (4.8)	*p* = 0.844
**Employment status**						
Employed	168 (84.4%)	153 (91.1%)	15 (8.9%)	χ^2^ = 1.501	5.6 (4.5)	F = 1.272
Unemployed	31 (15.6%)	26 (83.9%)	5 (16.1%)	*p* = 0.180	7.0 (6.0)	*p* = 0.199

M: mean; SD: standard deviation; N: sample; %: percentage; EPDS: Edinburgh postnatal depression scale; n.a.: not applicable. **In bold: significant values.** * Fisher’s exact test; ** Student’s *t*-test; *** ANOVA test.

**Table 2:** Clinical characteristics of the sample.

	Total Sample (N = 199) N (%)	EPDS- (N = 179) N (%)	EPDS+ (N = 20) N (%)	*p*-Value *	EPDS M (SD)	*p*-Value **
**16 < BMI < 18.49 Kg/m^2^**	18 (9.1%)	17 (94.4%)	1 (5.6%)	n.a.	6.3 (6.0)	F = 6.096 *p* = 0.901
**19 < BMI < 24.99 Kg/m^2^**	134 (67.3%)	122 (91%)	12 (9%)		5.8 (4.4)	
**25 < BMI < 29.99 Kg/m^2^**	29 (14.6%)	25 (86.2%)	4 (13.8%)		5.5 (5.8)	
**30 < BMI < 34.99 Kg/m^2^**	16 (8.0%)	14 (87.5%)	2 (12.5%)		5.6 (4.7)	
**BMI ≥ 35 Kg/m^2^**	2 (1.0%)	1 (50%)	1 (50%)		8.5 (4.9)	
**Family psychiatry history**						
none (N, %)	138 (69.3%)	132 (95.7%)	6 (4.3%)	χ^2^ = 16.194	4.7 (3.6)	F = 1.801
yes (N, %)	61 (30.7%)	47 (77.0%)	14 (23.0%)	***p* < 0.001**	8.2 (6.1)	***p* = 0.022**
**Physical comorbidities pre-pregnancy** (N, %)						
none	138 (69.3%)	127 (92%)	11 (8%)	χ^2^ = 2.153	5.2 (4.6)	F = 1.249
yes	61 (30.7%)	52 (85.2%)	9 (14.8%)	*p* = 0.114	7.0 (5.0)	*p* = 0.217
***Allergic diseases***						
none	15 (7.5%)	14 (93.3%)	1 (6.7%)	χ^2^ = 0.205	5.8 (4.8)	F = 0.879
yes	184 (92.5%)	165 (89.7%)	19 (10.3%)	*p* = 0.541	4.9 (4.2)	*p* = 0.619
***Respiratory diseases***						
none	193 (97%)	173 (89.6%)	20 (10.4%)	χ^2^ = 0.691	5.8 (4.8)	F = 0.541
yes	6 (3%)	6 (100%)	0 (0%)	*p* = 0.525	5.5 (2.9)	*p* = 0.950
***Neurological diseases***						
none	188 (94.5%)	170 (90.4%)	18 (9.6%)	χ^2^ = 0.852	5.7 (4.8)	F = 1.420
yes	11 (5.5%)	9 (81.8%)	2 (18.2%)	*p* = 0.305	7.5 (4.0)	*p* = 0.114
***Endocrinopathies***						
none	181 (91%)	166 (91.7%)	15 (8.3%)	χ^2^ = 6.880	5.7 (4.8)	F = 1.357
yes	18 (9%)	13 (72.2%)	5 (27.8%)	***p* = 0.022**	6.5 (4.9)	*p* = 0.146
***Cardiovascular diseases***						
none	190 (95.5%)	171 (90%)	19 (10%)	χ^2^ = 0.012	5.7 (4.8)	F = 0.740
yes	9 (4.5%)	8 (88.9%)	1 (11.1%)	*p* = 0.622	7.1 (3.1)	*p* = 0.787
***Neoplasia***						
none	193 (97%)	176 (91.2%)	17 (8.8%)	χ^2^ = 10.922	5.5 (4.4)	F = 5.604
yes	6 (3%)	3 (50%)	3 (50%)	***p* = 0.014**	14.8 (7.6)	***p* < 0.001**
***Gastrointestinal diseases***						
none	192 (96.5%)	172 (89.6%)	20 (10.4%)	n.a	5.8 (4.8)	F = 0.879
yes	7 (3.5%)	7 (3.9%)	0 (0%)		6.0 (2.3)	*p* = 0.618
***Gynecological diseases***						
none	193 (97%)	173 (89.6%)	0 (0%)	n.a.	5.8 (4.8)	F = 0.686
yes	6 (3%)	6 (3.4%)	20 (10.4%)		5.8 (4.1)	*p* = 0.843
**Pharmacotherapy pre-pregnancy**						
none (N, %)	129 (64.8%)	117 (90.7%)	12 (9.3%)	χ^2^ = 0.227	5.7 (5.1)	F = 0.100
yes (N, %)	70 (35.2%)	62 (88.6%)	8 (11.4%)	*p* = 0.402	5.9 (4.0)	*p* = 0.752
**TSH ≤ 0.3 mU/l** (N, %)	5 (2.5%)	5 (100%)	0 (0%)	n.a.	5.6 (3.9)	F = 0.729
**TSH ≥ 3.5 mU/l** (N, %)	29 (14.6%)	23 (79.3%)	6 (20.7%)		6.8 (5.4)	*p* = 0.483

M: mean; SD: standard deviation; N: sample; %: percentage; BMI: body mass index; EPDS: Edinburgh postnatal depression scale; TSH: thyroid-stimulating hormone; n.a.: not applicable. * Fisher’s exact test; ** ANOVA test. **In bold: significant values.**

**Table 3:** Gynecological-obstetric characteristics of the sample.

	Total Sample (N = 199) N (%)	EPDS- (N = 179) N (%)	EPDS+ (N = 20) N (%)	*p*-Value *	EPDS (M, SD)	*p*-Value **
**Previous pregnancy**						
none (N, %)	105 (52.8%)	98 (93.3%)	7 (6.7%)	χ^2^ = 2.815	6.0 (5.5)	F = 0.876
yes (N, %)	94 (47.2%)	81 (86.2%)	13 (13.8%)	*p* = 0.075	5.5 (3.9)	*p* = 0.621
**Previous miscarriage/** **induced abortion**						
none (N, %)	135 (67.8%)	125 (92.6%)	10 (7.4%)	χ^2^ = 3.243	5.1 (4.3)	F = 0.884
yes (N, %)	64 (32.1%)	54 (84.4%)	10 (15.6%)	*p* = 0.064	7.1 (5.4)	*p* = 0.612
**Medical assisted procreation**						
none (N, %)	193 (97%)	173 (89.6%)	20 (10.4%)	χ^2^ = 0.691	5.8 (4.8)	F = 0.538
yes (N, %)	6 (3%)	6 (100%)	0 (0%)	*p* = 0.525	5.5 (2.7)	*p* = 0.915
**Physical comorbidities** **during the pregnancy**						
none (N, %)	114 (57.3%)	106 (93%)	8 (7%)	χ^2^ = 2.715	5.1 (4.3)	F = 0.907
yes (N, %)	85 (42.7%)	73 (85.9%)	12 (14.1%)	*p* = 0.080	6.7 (5.2)	*p* = 0.583
***Gestational hypertension/*** ***preeclampsia***						
none (N, %)	183 (92%)	166 (90.7%)	17 (9.3%)	χ^2^ = 1.457	5.6 (4.5)	F = 2.010
yes (N, %)	16 (8%)	13 (81.3%)	3 (18.8%)	*p* = 0.207	8.3 (6.6)	***p* = 0.008**
***Gestational Diabetes***						
none (N, %)	174 (87.4%)	158 (90.8%)	16 (9.2%)	χ^2^ = 1.120	5.6 (4.8)	F = 1.358
yes (N, %)	25 (12.6%)	21 (84.0%)	4 (16%)	*p* = 0.230	7.2 (4.7)	*p* = 0.145
***Shorten of uterus neck***						
none (N, %)	190 (95.5%)	173 (91.1%)	17 (8.9%)	χ^2^ = 5.653	5.5 (4.4)	F = 3.931
yes (N, %)	9 (4.5%)	6 (66.7%)	3 (33.3%)	***p* = 0.049**	10.9 (8.4)	***p* < 0.001**
***Miscarriage threats/*** ***placental abruption***						
none (N, %)	190 (95.5%)	172 (90.5%)	18 (9.5%)	χ^2^ = 1.545	5.7 (4.8)	F = 1.839
yes (N, %)	9 (4.5%)	7 (77.8%)	2 (22.2%)	*p* = 0.225	7.2 (4.1)	***p* = 0.018**
***Hematic/amniotic lost***						
none (N, %)	185 (93%)	166 (89.7%)	19 (10.3%)	χ^2^ = 0.141	5.8 (4.7)	F = 0.982
yes (N, %)	14 (7.0%)	13 (92.9%)	1 (7.1%)	*p* = 0.578	5.1 (6.2)	*p* = 0.488
***Dysthyroidism***						
none (N, %)	176 (88.4%)	158 (89.8%)	18 (10.2%)	χ^2^ = 0.053	5.8 (4.9)	F = 0.798
yes (N, %)	23 (11.6%)	21 (91.3%)	2 (8.7%)	*p* = 0.585	5.5 (3.9)	*p* = 0.720
***Genitourinary infection***						
none (N, %)	185 (93%)	166 (89.7%)	19 (10.3%)	χ^2^ = 0.141	5.8 (4.8)	F = 0.744
yes (N, %)	14 (7%)	13 (92.9%)	1 (7.1%)	*p* = 0.578	5.4 (3.9)	*p* = 0.783

N: sample; %: percentage; EPDS: Edinburgh postnatal depression scale; * Pearson’s 2 test—Fisher’s exact test; ** Student’s *t*-test. **In bold: significant values.**

**Table 4:** Delivery-related characteristics of the postpartum sample.

	Puerperal Women (N = 144)	EPDS- (N = 130)	EPDS+ (N = 14)	EPDS (M, SD)	*p*-Value *^,^**
**Preterm birth**					
none (N, %)	107 (74.3%)	95 (88.8%)	12 (11.2%)	5.7 (4.9)	* t = −0.098
yes (N, %)	37 (25.7%)	35 (94.6%)	2 (5.4%)	5.8 (4.3)	*p* = 0.922
**Post-term birth**					
none (N, %)	88 (61.1%)	80 (90.9%)	8 (9.1%)	6.0 (5.1)	* t = 1.064
yes (N, %)	56 (38.9%)	50 (89.3%)	6 (10.7%)	5.2 (4.3)	*p* = 0.289
**Cesarean delivery**					
none (N, %)	92 (63.9%)	83 (90.2%)	9 (9.8%)	5.4 (4.6)	* t = 1.153
yes (N, %)	52 (36.1%)	47 (90.4%)	5 (9.6%)	6.3 (5.2)	*p* = 0.251
**APGAR, 5 min**					
regular (N, %)	132 (91.7%)	119 (90.2%)	13 (9.8%)	5.7 (4.9)	* t = −0.209
irregular (N, %)	12 (8.3%)	11 (91.7%)	1 (8.3%)	5.4 (3.9)	*p* = 0.835
**APGAR, 10 min**					
regular (N, %)	131 (91%)	118 (90.1%)	13 (9.9%)	5.7 (4.9)	* t = −0.303
irregular (N, %)	13 (9%)	12 (92.3%)	1 (7.7%)	5.3 (3.8)	*p* = 0.762
**Low birth weight**					
none (N, %)	124 (86.1%)	112 (90.3%)	12 (9.7%)	5.5 (4.8)	* t = 1.364
yes (N, %)	20 (13.9%)	18 (90%)	2 (10%)	7.1 (4.8)	*p* = 0.175
**Newborn cranial** **circumference**					
low (N, %)	47 (32.6%)	43 (91.5%)	4 (8.5%)	5.7 (5.1)	** F = 0.065 *p* = 0.937
regular (N, %)	91 (63.2%)	81 (89%)	10 (11%)	5.7 (4.8)	
large (N, %)	6 (4.2%)	6 (4.6%)	0 (0%)	5 (3.5)	
**Newborn length**					
low (N, %)	4 (2.8%)	4 (100%)	0 (0%)	3.5 (4.4)	** F = 0.428 *p* = 0.652
regular (N, %)	78 (54.2%)	71 (91%)	7 (9%)	5.8 (4.8)	
large (N, %)	62 (43.1%)	55 (88.7%)	7 (11.3%)	5.7 (4.9)	
**Type of breastfeeding**					
maternal (N, %)	82 (56.9%)	73 (89%)	9 (11%)	5.7 (4.8)	** F = 0.428 *p* = 0.653
artificial (N, %)	12 (8.3%)	10 (83.3%)	2 (16.7%)	6.8 (7.5)	
mixed (N, %)	50 (34.7%)	47 (94%)	3 (6%)	5.4 (4.1)	

N: sample; %: percentage; EPDS: Edinburgh postnatal depression scale; * Fisher’s Exact test; ** ANOVA test.

**Table 5:** Multiple linear regression analysis (outcome = EPDS total score).

Predictors	t	B	SE	95% CI (B)	*p*
**WQ**	0.146	0.107	0.731	(−1.335)–(1.549)	0.884
**Stress Scale HR**	4.353	2.465	0.566	(1.348)–(3.583)	**<0.001**
**GHQ**	−0.065	−0.074	1.147	(−2.338)–(2.189)	0.948
**Foreign nationality**	−3.046	−2.954	0.970	(−4.866)–(−1.041)	**0.003**
**Familiar positive** **psychiatric history**	−3.616	−2.338	0.646	(−3.613)–(−1.063)	**<0.001**
**Neoplastic diseases**	−3.645	− 6.548	1.796	(−10.092)–(−3.005)	**<0.001**
**Gestational** **hypertension/preeclampsia**	−1.337	−1.453	1.087	(−3.596)–(−0.691)	0.183
**Miscarriage threats/** **placental abruption**	−0.098	−0.140	1.426	(−2.954)–(2.673)	0.922
**Shortening of uterus neck**	−0.817	−1.215	1.488	(−4.150)–(1.719)	0.415

B: coefficient of not standardized regression; SE: standard error; CI: confidence interval; *p*: *p*-value of significance. **In bold: significant values.**

## Data Availability

The data presented in this study are available on request from the corresponding author.
